# Living Arrangements and Psychosocial Well-Being Among Older Adults: Evidence From the Health and Retirement Study

**DOI:** 10.1093/geroni/igab046.465

**Published:** 2021-12-17

**Authors:** Rashmita Basu, Huabin Luo, Adrienne Steiner, Alan Stevens

**Affiliations:** 1 East Carolina University, Greenville, North Carolina, United States; 2 East Carolina University, East Carolina University, North Carolina, United States; 3 Baylor Scott & White Health Research Institute, Temple, Texas, United States

## Abstract

Despite growing attention to the association between living arrangements and health outcomes, less is known about how emotional well-being and life satisfaction vary by living arrangements. Using data from the 2014 and 2016 Leave Behind Questionnaires from the Health and Retirement Survey (N=13,275), we estimated generalized linear regression models comparing emotional well-being (a ratio of positive to negative emotion) and life satisfaction (the satisfaction with life scale, SWLS) by living alone versus living with others, controlling for socioeconomic and other health-related characteristics. Overall, individuals who lived alone had lower emotional well-being (β=-0.11; p<0.01), and SWLS score (β=-0.42; p<0.001), compared to those living with others. The direction of these relationships stratified by the cognitive status was the same. Policies and programs designed to support the growing population of older adults living alone should focus on improvement in these positive outcomes to enhance the quality of life.

